# Grip control and motor coordination with implanted and surface electrodes while grasping with an osseointegrated prosthetic hand

**DOI:** 10.1186/s12984-019-0511-2

**Published:** 2019-04-11

**Authors:** Enzo Mastinu, Francesco Clemente, Paolo Sassu, Oskar Aszmann, Rickard Brånemark, Bo Håkansson, Marco Controzzi, Christian Cipriani, Max Ortiz-Catalan

**Affiliations:** 1 Department of Electrical Engineering, Chalmers University of Technology, Gothenburg, Sweden; 2 The Biorobotics Institute, Scuola Superiore Sant’Anna, Pisa, Italy; 3 Prensilia SRL, Pisa, Italy; 4 Department of Hand Surgery, Sahlgrenska University Hospital, Gothenburg, Sweden; 5 Christian Doppler Laboratory for Restoration of Extremity Function, Department of Surgery, Medical University of Vienna, Vienna, Austria; 6 Department of Orthopaedics, Gothenburg University, Gothenburg, Sweden

**Keywords:** Prosthetic control, Osseointegration, Electromyography (EMG), Implanted electrodes, Epimysial

## Abstract

**Background:**

Replacement of a lost limb by an artificial substitute is not yet ideal. Resolution and coordination of motor control approximating that of a biological limb could dramatically improve the functionality of prosthetic devices, and thus reduce the gap towards a suitable limb replacement.

**Methods:**

In this study, we investigated the control resolution and coordination exhibited by subjects with transhumeral amputation who were implanted with epimysial electrodes and an osseointegrated interface that provides bidirectional communication in addition to skeletal attachment (e-OPRA Implant System). We assessed control resolution and coordination in the context of routine and delicate grasping using the Pick and Lift and the Virtual Eggs Tests. Performance when utilizing implanted electrodes was compared with the standard-of-care technology for myoelectric prostheses, namely surface electrodes.

**Results:**

Results showed that implanted electrodes provide superior controllability over the prosthetic terminal device compared to conventional surface electrodes. Significant improvements were found in the control of the grip force and its reliability during object transfer. However, these improvements failed to increase motor coordination, and surprisingly decreased the temporal correlation between grip and load forces observed with surface electrodes. We found that despite being more functional and reliable, prosthetic control via implanted electrodes still depended highly on visual feedback.

**Conclusions:**

Our findings indicate that incidental sensory feedback (visual, auditory, and osseoperceptive in this case) is insufficient for restoring natural grasp behavior in amputees, and support the idea that supplemental tactile sensory feedback is needed to learn and maintain the motor tasks internal model, which could ultimately restore natural grasp behavior in subjects using prosthetic hands.

## Introduction

The last decade has witnessed significant progress in the field of upper limb prosthetics. A deeper understanding of basic scientific questions in neurophysiology and neuroscience met novel surgical techniques and prosthetic components, such as articulated hands and myo-controllers that reached the market [1]. However, although myoelectric prostheses have increased dexterity and anthropomorphism, the control interface, namely surface electromyography (sEMG), has remained basically unchanged in the last four decades [2–4]. This interface entails applying electrodes on the skin of the amputee’s stump to capture the electrical activity of residual muscles (myoelectric signal) and translate it into movements of the prosthesis [5]. Amputees therefore must learn to contract their residual muscles to control prosthetic devices. This control approach is effective due to its simplicity but, unfortunately, the limited stability of signals recorded over the skin curtails its reliability. Factors such as temperature, electromagnetic interference, body impedance changes, and motion artefacts can have a destructive effect on the reliability of sEMG-based prosthetic devices.

Implanted myoelectric electrodes are candidates for solving this issue, developed on the principle that a more intimate connection with the information source allows for collection of volitional signals in a more selective and reliable fashion [6–8]. Since the 1970s, several groups have demonstrated and clinically assessed various technological approaches, including intramuscular and epimysial electrodes [9–11]. Whereas the differences between implantable and sEMG electrodes have been investigated in terms of signal to noise ratio, selectivity, and stability [5], only a few studies compared them from the perspective of an interface for prosthetic control [11–13]. Notably, none explored their effects on functional tasks. In this study we endeavored to bridge this gap.

Three transhumeral amputees who received the e-OPRA Implant System (Integrum AB, Sweden) participated in this study. The e-OPRA is an osseo-neuromuscular interface that combines osseointegration for direct skeletal attachment with implanted neuromuscular electrodes for control and sensory feedback [11]. We compared the control performance of these subjects while using a prosthesis under myoelectric control by either surface electromyography (sEMG) or implanted epimysial electromyography (eEMG). The Virtual Eggs Test (VET) [14] and Pick and Lift Test (PLT) [15] were used to evaluate grip force control and motor coordination in the context of delicate and routine grasping.

The VET required the subject to pick and transfer fragile objects. It demanded fine grasping control skills and a reliable interface, because excessive *grip forces*, involuntary EMG signals, or EMG artifacts, could break the objects. The PLT measured the motor coordination, i.e. the ability to coordinate *grip forces* (GF) and *load forces* (LF) while lifting an object and, again, the reliability of the recorded myoelectric signal while transporting the object. Motor coordination is known as a distinctive feature in mature grasping in healthy humans, and as such, a comparable behavior is desired by users of prosthetic hands.

Sensory feedback is believed crucial to building and continuously maintaining a functioning motor control repertoire in humans [16–18]. In this study, subjects had at their disposal sensory feedback in the form of vision and osseoperception (mediated by both hearing and touch) [19]. Despite the lack of somatosensory feedback other than osseoperception, we deemed that the available task-relevant incidental feedback would suffice to restore near-natural motor control skills. Saunders et al. [20] and Hermsdörfer et al. [18] found that when highly reliable efferent signals are available for control, incomplete afferent information may be still enough to retain the motor control library. Hence, assuming a superior controllability offered by the epimysial electrodes, we hypothesized that the eEMG controller would entail better (i) grip force control, (ii) reliability, and (iii) motor coordination than the sEMG.

## Materials and methods

### Subjects

Three subjects with transhumeral amputation where recruited to participate in this study (hereafter referred to as S1, S2 and S3). Subjects S1 and S2 were implanted with e-OPRA in 2017, whereas S3 was implanted previously in 2013. Additionally, S1 and S2 underwent a *Targeted Muscle Reinnervation* (TMR) surgical procedure for redirecting the radial nerve into the lateral head of the triceps brachii muscle, and the ulnar nerve into the short head of the biceps brachii muscle, aiming for intuitive myoelectric signals for hand open and close, respectively [21, 22]. Bipolar epimysial electrodes were implanted on these TMR reconstructions as well as on the naturally innervated long head of the triceps brachii muscle and on the long head of the biceps brachii muscles. The implanted electrodes were accessed via the e-OPRA Implant System. The tests were conducted between February 2018 and May 2018. The study was approved by the Swedish regional ethical committee in Gothenburg (Dnr: 769–12).

### Materials

The subjects performed the VET and PLT while operating their daily transhumeral prostheses that were composed of a myoelectric-locking elbow and a myoelectric terminal device (12 K50 Elbow and VaryPlus Hand, Ottobock, Germany). The terminal device was controlled using the conventional direct (one-for-one) and proportional control strategy [23], fed by either sEMG or eEMG signals. Concerning the sEMG configuration, the two muscle sites were optimally targeted with MyoBock electrodes (Ottobock, Germany) connected directly to the terminal device. This configuration represents the most common solution for myoelectric control in clinical practice. The eEMG configuration used the signals from epimysial electrodes amplified and filtered by a custom-designed embedded system contained within the prosthetic device [24]. The algorithm implemented for the one-for-one control resembled the behavior of conventional MyoBock electrodes. The implementation was based on the direct mapping of the speed of each prosthetic hand movement to the Mean Absolute Value of its corresponding channel, calculated from 50 ms non-overlapping windows of eEMG data sampled at 500 Hz [25, 26].

For each subject, the muscles used for control were the same for both sEMG and eEMG configurations. In the case of S1 and S2, the TMR reconstructions on triceps and biceps muscles were voluntary contracted by respectively opening and closing the phantom hand, these contractions were then directly mapped to the corresponding movement of the prosthetic hand. For S3, the contractions of the naturally innervated portion of triceps and biceps were directly mapped to open and close the prosthesis, as this subject has done daily for the last decade. The prosthesis was, for both control configurations, mechanically attached to the subject’s stump via a clamp mechanism over the percutaneous portion of the osseointegrated implant.

### Experimental protocol

**Fig. 1:**
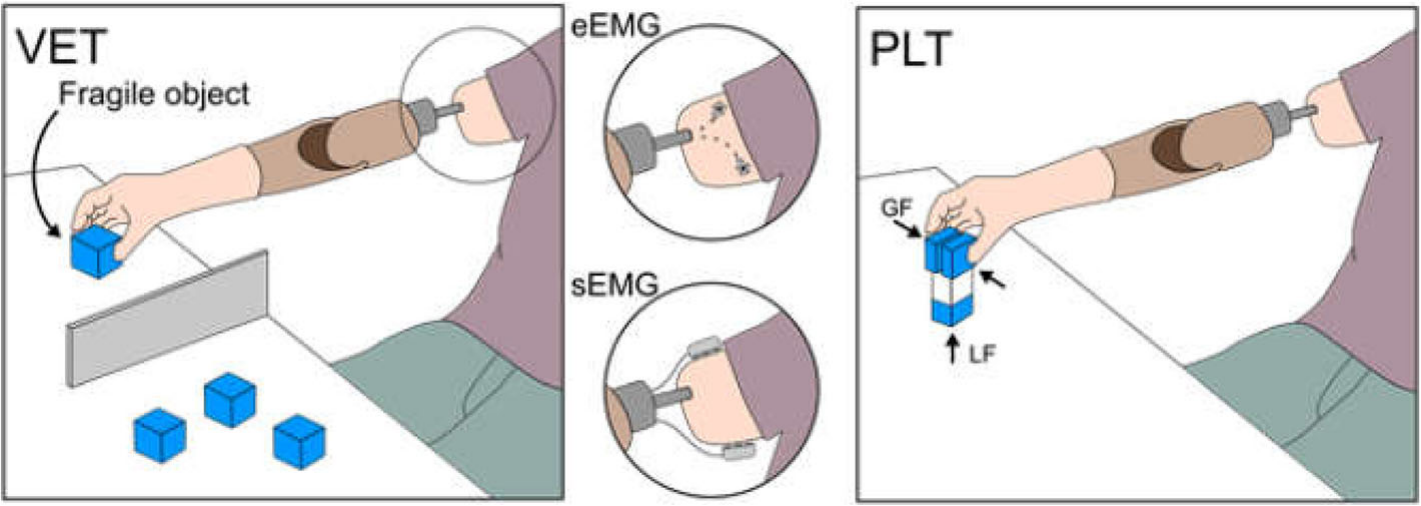
Experimental setup for the Virtual Eggs Test (VET, left) and the Pick and Lift Test (PLT, right). Both tests were performed while the subjects controlled their prosthesis through epimysial electrodes (eEMG configuration) and surface electrodes (sEMG configuration)

The subjects performed the VET and PLT with either sEMG or eEMG configurations (Fig. 1). Both tests were performed while seated in front of a table. The chair height and its distance from the table were adjusted for each subject to achieve a comfortable position to interact with the test objects. The prosthetic elbow joint was kept in a fully extended position during the entire experiment.

The VET was used to measure the subjects’ ability to regulate *grip force*, which is also affected by the reliability of the interface, while maneuvering the prosthesis in the context of delicate grasping (i.e. when handling fragile objects). The VET was first presented by Clemente et al. as a modification of the well-known box and blocks test for gross manual dexterity [14, 27], and resembles a task of picking and repositioning fragile objects without breaking them. Here, 50 × 50 × 50 mm^3^ plastic blocks weighing 55 g were equipped with a magnetic fuse, using a magnetic latching mechanism placed in between the opposite walls of the block. A force applied on the walls exceeding a fixed threshold caused the fuse to break instantaneously, similarly to “breaking an egg”. As the VET prescribes, the subjects were asked to transfer the blocks from one side of a plastic wall (height of 15 cm) to the other, as quickly as possible within one minute while also preventing their breakage. The subjects were instructed to complete the action of transferring a block even if this broke while grasping it or during transfer. The number of broken blocks and the total number of transferred blocks (thus comprising both the blocks that remained intact and that broke) were measured. The subjects were asked to perform ten one-minute sessions, half of which were performed with blocks having thresholds set at 18 ± 0.2 N (mean ± sd, VET_18N_) and half of which with blocks having thresholds set at 6 ± 0.2 N (VET_6N_). Before the evaluation, all subjects performed a single training session to become accustomed with the task.

Picking and lifting an instrumented object was used to assess the subjects’ motor coordination as well as the reliability of the interface when maneuvering the prosthesis in the context of routine grasping (PLT) [15, 28]. The test object consisted of a 40 × 45 × 130 mm^3^ plastic block (~ 200 g) with three embedded load cells (SMD2551, Strain Measurements Devices, UK), of which two measured the *grip force* of the thumb and fingers independently and the third measured the *load force* applied on the object before lift-off. The test consisted of five series of 20 repetitions each (100 repetitions in total), performed in a single experimental session. Each repetition was performed at self-selected speed and consisted of: 1) moving the arm to reach the object, 2) grasping the object, 3) lifting the object a few centimeters above the desk, 4) repositioning the object back on the table and 5) releasing the object. The grasped surfaces of the test object were covered with sandpaper. The coefficient of friction between the object and the prosthesis was found to be 0.9. Consequently, the minimum *grip force* required to lift the object was 1.9 N, corresponding to a minimum grip/load force ratio (i.e. the slip GLF_r_) of 1.1. A repetition was deemed successful if it was performed without overcoming a predefined limit on *grip force*, set to 30 N (GF_THRESH_ –software break signaled to the subjects by audio-visual feedback), corresponding to a GLF_r_ of 16. The *grip force* limit was set empirically after pilot experiments. The limit was introduced to prevent the subjects adopting a strategy in which they grasped the object by fully closing the prosthetic digits. Each subject performed a short training session comprising 10 repetitions.

### Data analysis and statistical methods

In the VET, the number of broken and transferred blocks were compared with regards to control configurations (sEMG and eEMG) and used as an assessment for *grip force* control. In particular, we computed the probability of the observed occasions in which the number of broken or transferred blocks in the eEMG configuration was different than in the sEMG configuration for all subjects assuming a binominal distribution (i.e., *B*(*x*; *n*, *p*) with *x* equal to the number of successes, *n* equal to 3 corresponding to the number of subjects in the group, and *p* = 0.5 (assuming that the two events have the same probability to happen), akin to Clemente et al. [14].

In the PLT, motor coordination was evaluated through the level of *grip-load force* (GF-LF) coordination, quantified through the temporal delay between the instants when the GF and LF reached 50% of the LF at lift-off. In addition, we calculated two other performance metrics from the PLT, namely the maximum grip force during a repetition (GF_MAX_), and the difference between the grip force at lift-off and GF_MAX_ during the holding phase (ΔGF). Specifically, the GF_MAX_ provided an indication of the subjects’ grasp force instinctively produced at lift-off, whereas the ΔGF was related to potentially involuntary changes in the grip force during hold (i.e., owing to cross-talk or motion artifacts). As such, lower GF_MAX_ and ΔGF values are considered to indicate better performance. These metrics provide insights about both grip force control and reliability of the interface, two aspects that should be considered together as a trustworthy assessment of control is only possible when a reliable interface is available. The Wilcoxon rank-sum test was used to compare these performance metrics across configurations.

In all cases, a *p* value lower than 0.05 was considered as reference for statistical significance. We would like to point out that these commonly used statistical methods were used to analyze the results of the study for scientific rigor. Albeit useful, in this case the information on statistical significance is limited because of the limited number of subjects involved in the study. The restricted availability of subjects recipient of the e-OPRA Implant System limited our possibility to carry out a larger study. For these reasons, in the following we present and discuss the results in a descriptive fashion.

## Results

**Fig. 2:**
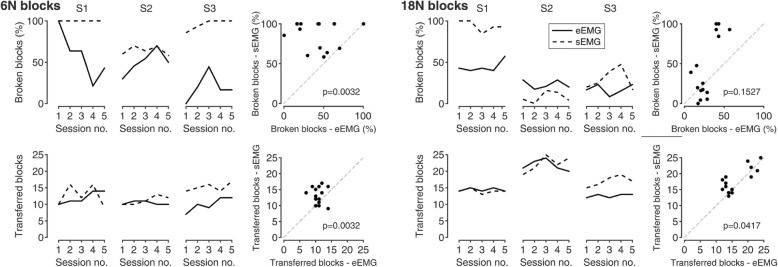
Virtual Eggs Test results for 6 N (left) and 18 N (right) blocks in terms of number of broken (top row) and transferred blocks (bottom row). The transferred blocks are shown as absolute number while the broken blocks are shown as percentage of the transferred blocks. Solid and dashed lines represent eEMG and sEMG configurations, respectively. The scatter plots compare the transferred and broken blocks between the two configurations. Each dot represents a single experimental session from one subject. In the VET_6N_, subjects broke fewer blocks in the eEMG configuration in 13 out of 15 sessions (3 subjects × 5 sessions), considerably improving their performance when using epimysial electrodes. On the other hand, more blocks were transferred when using the sEMG in 10 out of 15 total sessions. Similarly, in the VET_18N_, subjects broke fewer blocks in the eEMG configuration in 9 out of 15 sessions but transferred more blocks in sEMG configuration in 8 out of 15 sessions

In the VET_6N_, subjects broke fewer blocks in the eEMG configuration in 13 out of 15 sessions (3 subjects × 5 sessions), considerably improving their performance when using epimysial electrodes (*p* = 0.003, Fig. 2). Overall, all subjects performed better when using the eEMG than the sEMG control. Two out of three subjects (S1 and S3) broke 100% of the transferred blocks in the majority of the performed sessions (7 out of 10) under sEMG. On the other hand, more blocks were transferred when using the sEMG in 10 out of 15 sessions (p = 0.003), albeit blocks were considered transferred even when broken.

In the VET_18N_, subjects broke fewer blocks in the eEMG configuration in 9 out of 15 sessions (*p* = 0.153). Similar to VET_6N,_ the subjects transferred more blocks in sEMG configuration than eEMG in 8 out of 15 sessions (*p* = 0.042).

No strong differences in the number of transferred blocks were observed between VET_6N_ and VET_18N_ (Fig. 2, second row), as only S2 improved performance consistently (from ~ 10 to > 20 transferred blocks). In terms of broken blocks, the subjects during the sEMG configuration performed better when handling 18 N blocks compared to 6 N blocks (from 60 to 10% for S2, from ~100% to 20–40% for S3). A trend was observed for S3 in the number of transferred blocks for VET_6N_ and VET_18N_. This subject transferred more blocks during the sEMG configuration for both tests.

**Fig. 3:**
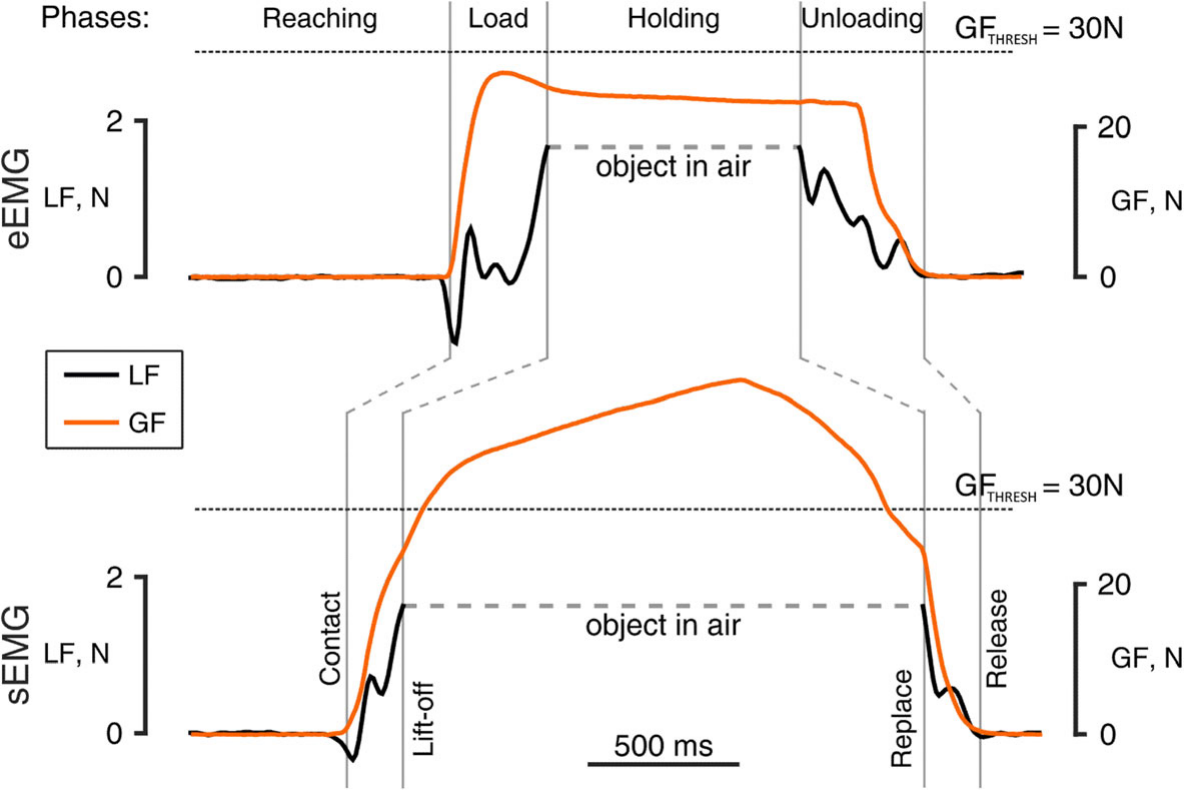
Representative pick-and-lift repetition for the eEMG (top) and the sEMG (bottom) configurations. The top grey labels represent the manipulation task phases, while the vertical grey lines represent the key mechanical events delimiting task phases. In the eEMG configuration, subjects typically showed more control over the grip force applied on the object: less force produced and a more stable grip during the holding phase

**Fig. 4:**
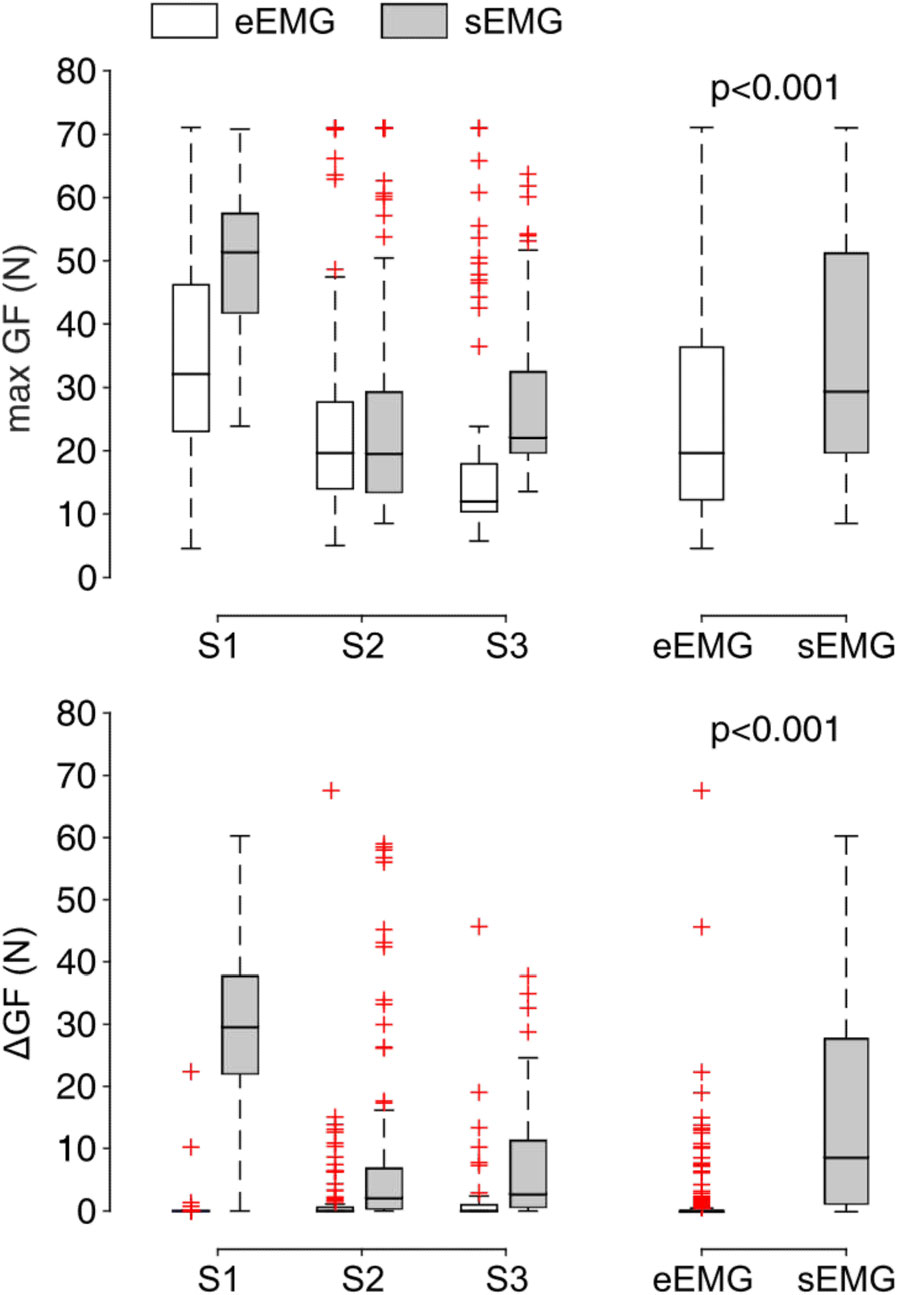
Grip Force (GF) analysis from the pick and lift task for eEMG and sEMG configurations: maximum GF (top) and GF change during holding phase (bottom). The GF change was calculated as the difference between the maximum GF in the holding phase and the GF at lift-off. The maximum GF and the GF change during holding phase were considerably larger for the sEMG configuration (*p* = 0.001)

**Fig. 5:**
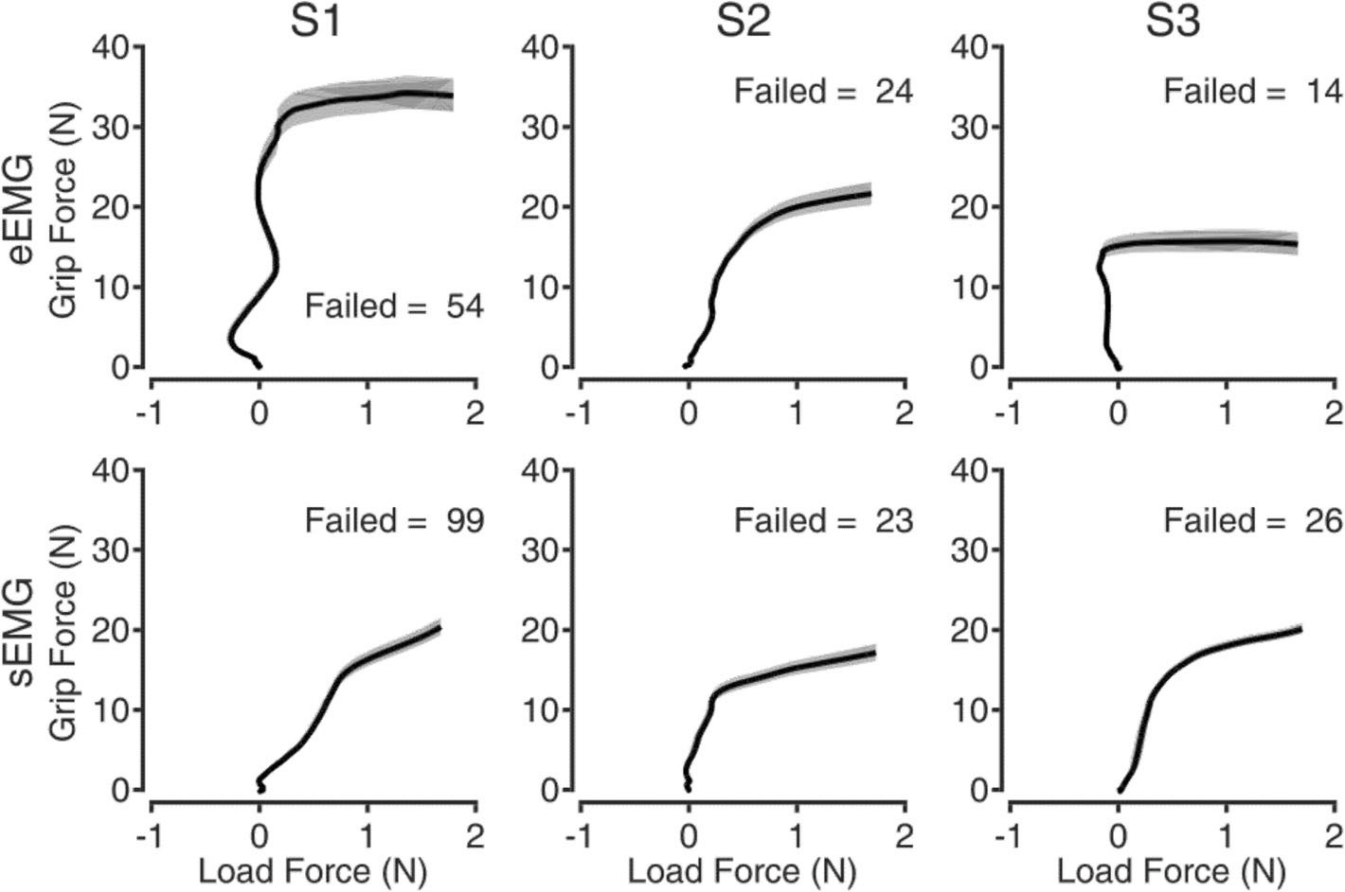
Motor coordination during the pick and lift task for the eEMG (top) and sEMG (bottom) configurations. Grip force vs load force profiles from object contact to lift-off for all subjects and test configurations. None of the configurations promoted coordinated grip-load forces patterns in subjects’ performance. Unexpectedly, sEMG curves revealed a more linear ratio, closer to the mature grasp behavior observed in able-bodied adult humans, even though the instrumented object virtually broke (task failure) considerably more frequently in the sEMG than in the eEMG configuration for S1 and S3

The PLT showed interesting results (Fig. 3). When using eEMG, subjects typically showed more control over the *grip force* applied on the object. In particular, *grip force* during the holding phase resembled the force profile applied by able-bodied subjects, i.e. it peaked at lift-off and remained constant until the object was repositioned on the table [16] (Fig. 3). This was not the case for the sEMG configuration, where *grip force* continued to increase even during the holding phase. Consequently, a significant increase in the ΔGF was found between the sEMG and the eEMG configurations (*p* < 0.001, Fig. 4), from 0:0.24 N (median: IQR) to 8.66:26.51 N. A significant difference was found also in the GF_MAX_ (p < 0.001, Fig. 4), with *grip force* considerably larger in the sEMG (29.42:31.52 N) with respect to the eEMG configuration (19.64:24.16 N). This is also reflected in the safety margin (i.e. the difference between the employed GFL_r_ and the slip GFL_r_ [15]), which was found to be 15 and 20 for the eEMG and sEMG configuration, respectively. The instrumented object virtually broke (task failure) more frequently in the sEMG than in the eEMG configuration (49% versus 31%, respectively, Fig. 5).

**Fig. 6:**
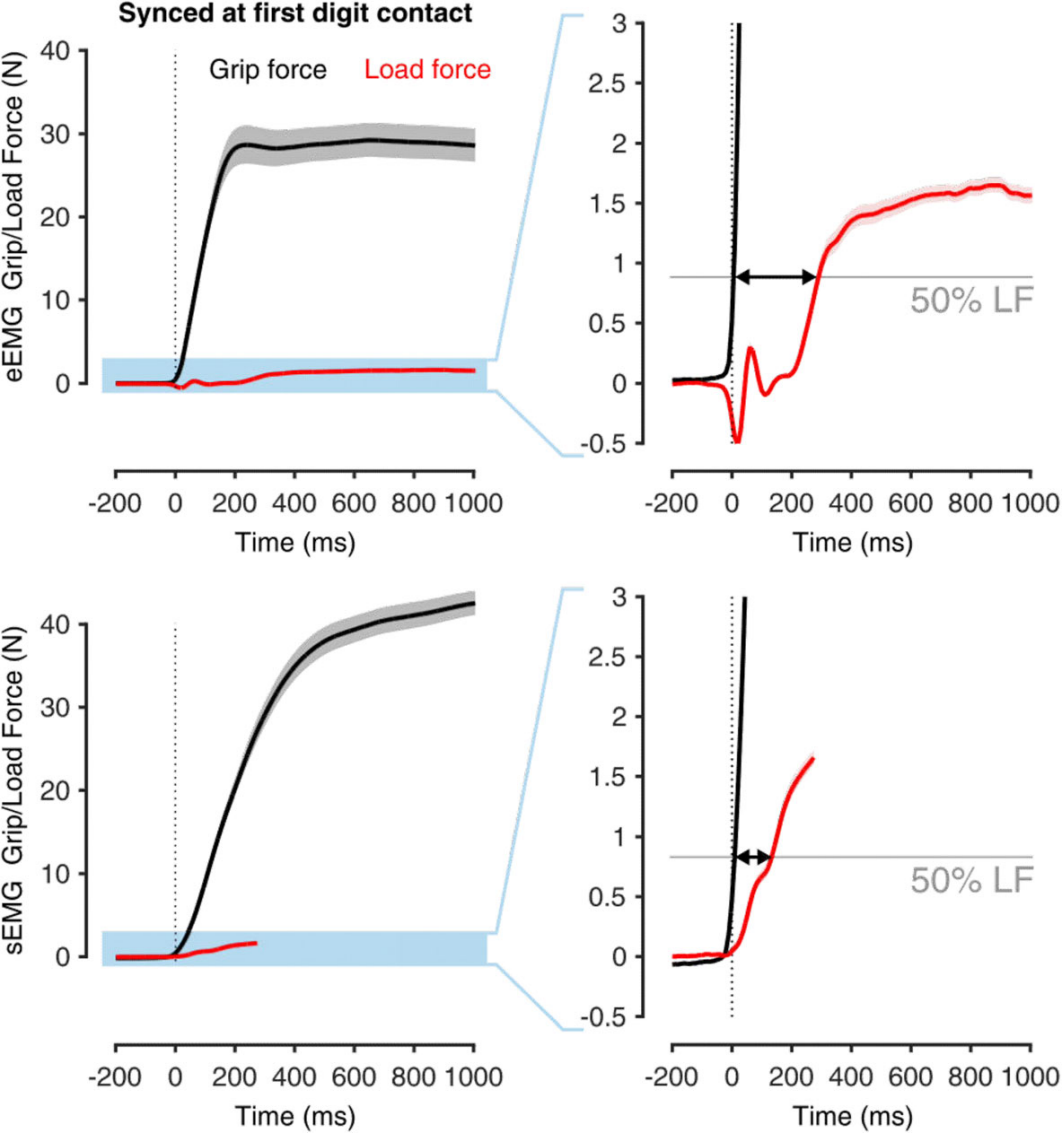
Motor coordination during the pick and lift task for eEMG (top) and sEMG (bottom) configurations for S1. The temporal delay between the instants when the grip and load forces reached 50% of the load force at lift-off significantly reduced from 297:160 ms (median: IQR) in the eEMG configuration to 183:118 ms in the sEMG configuration (*p* < 0.001). Namely, the median delay was reduced by 39% when surface electrodes were used

None of the configurations promoted coordinated GF-LF patterns in subjects’ performance. Unexpectedly, the subjects showed greater motor coordination in the sEMG compared to the eEMG configuration. The sEMG GF and LF appeared more temporally correlated, closer to the mature grasp behavior observed in able-bodied adult humans [28] (Fig. 5). More quantitatively, the temporal delay between the instants when the GF and LF reached 50% of the LF at lift-off significantly reduced (*p* < 0.001) from 297:160 ms in the eEMG configuration to 183:118 ms (Fig. 6). Namely, the median delay was reduced by 39% when surface electrodes were used. In addition, the load phase duration decreased as well from 360:205 ms in the eEMG configuration to 250:130 ms in the sEMG configuration (*p* < 0.001).

## Discussion

In this study, we compared implanted epimysial (eEMG) and conventional surface (sEMG) electrodes in terms of functional prosthetic control within routine and delicate grasp tasks. In particular, we hypothesized that eEMG-based control would entail better (i) grip force control, (ii) reliability, and (iii) motor coordination than sEMG-based control. The comparison was planned on a group of three transhumeral amputee subjects with osseointegrated prostheses (e-OPRA Implant System). To the best of our knowledge, these are the only subjects with chronically implanted epimysial electrodes and therefore we had a limited number of subjects for this experiment. It is important to mention that we could not mask to the subjects which configuration, implanted or surface electrodes, was currently in use during the tests. Therefore, the potential bias of these subjects towards the e-OPRA technology, to which clinical trial they volunteered to participate, should be considered.

It is also worth mentioning that two out of the three participants underwent *Targeted Muscle Reinnervation* surgery. For these subjects, the TMR sites were used to actuate the prosthesis during the tests, as well as routinely in their daily life. This means that, for these subjects, the opening or closing of the prosthetic hand was produced by intuitive contractions of muscles, rather than counterintuitive contractions on naturally innervated biceps and triceps muscles as conventionally done for transhumeral amputees. These subjects treated with e-OPRA and TMR have repeatedly emphasized the importance of reliable prosthetic movements which are felt as intuitive or “natural”. This study provides evidence on the successful combination of implanted electrodes and TMR surgery for transhumeral amputees. However, we could not observe or distinguish any difference between the subjects with and without TMR in the particular tasks we evaluated.

eEMG resulted in better grip force control at a cost. As measured by the VET_6N_ (lower number of broken blocks, Fig. 2), this study suggests that implanted epimysial electrodes improve grip force control of a prosthetic hand over surface EMG. However, the number of transferred blocks was not consistent with this higher precision since fewer blocks were transferred under eEMG. One must keep in mind that considering speed of execution in relation to the number of transferred blocks can be misleading since broken blocks also counted as transferred. Even if the larger control reliability of eEMG was exploited to grasp the fragile objects, the resulting control strategy still highly depended on visual feedback, as could be argued from the longer load phase duration in the PLT. Attention to visual feedback operating at higher resolution could have caused a slower operation as opposed to sEMG where a more feedforward strategy was employed. This interpretation is supported by the PLT results, where the subjects showed improved coordination between grip and load forces. It is of interest that this difference in execution speed was also observed in the VET_18N_ but without significant differences in the number of broken blocks, meaning that the more feedforward approach of sEMG was actually advantageous in that context (gross grasping). Indeed, the subjects’ performance in the VET_18N_ improved much more consistently for sEMG than eEMG. Interestingly, the subjects in the eEMG configuration did not adapt their behavior to their arguably improved controllability, probably because they still prioritized trying to reduce the number of broken blocks as much as possible.

Both the VET and the PLT showed that sEMG led to a significantly higher grip force applied during object manipulation (higher number of broken blocks for the VET_6N_ and failed trials for the PLT, higher GF_MAX_ and safety margin). However, we hypothesize that the larger force produced might be simply related to the poor reliability of the sEMG interface and consequently higher variability of the prosthesis dynamic behavior, and not much can be said about tasks being performed more economically with eEMG. This poorer grip force control with sEMG seems to contrast the results from Markovic and colleagues [29], who reported that incidental feedback is sufficient to regulate grip force. However, their results were obtained in a rather different setup from our experimental conditions (e.g. prosthesis detached from the body, arm in a rest position, healthy subjects, no functional task carried out). This discrepancy underlines the importance of testing myoelectric prosthesis control in the context of functional tasks.

Higher reliability of the eEMG interface was confirmed. In normal sensorimotor conditions, *grip force* remains stable between lift-off and reposition [16]. This was the case for the eEMG but not for the sEMG configuration. In the latter, the *grip force* consistently increased soon after the lift-off (Fig. 3), resulting into a larger number of failed trials (Fig. 5). A larger *grip force* than necessary can be intuitively associated to increased muscular effort from the subject, and consequently to a less economized performance of the task. However, this is not necessarily true when the control interface is as unreliable as is typically experienced by sEMG control users. In fact, any artifact produced from neighboring muscles, a sudden environmental interference or shift of electrode position could elicit unwanted activation of the prosthesis, causing unintended application of significant force to the grasped object. We argue that the larger ΔGF observed in the sEMG condition is due to the shoulder muscles activation. This condition is a well-known limitation of surface myoelectric control approach for transhumeral amputees, making it difficult for subjects to interact with objects placed higher than chest-level [11]. Contrastingly, the eEMG interface circumvented this problem.

Despite its finer grip force control and the higher reliability, eEMG control failed to promote better motor coordination. Many studies assessed GF vs LF coordination during pick-and-lift tasks, and literature widely agrees on the concurrent increase in GF and LF before lift-off [16, 30]. It is also widely accepted that impairment of this grip-load force coupling can be found in subjects with disrupted cutaneous afferent feedback, such as that caused by anesthetizing the fingertips [15, 31], as well as in subjects with inconsistent tactile feedback [32, 33]. Amputees lack of any tactile sensory feedback, and their myoelectric control is mainly regulated by visual feedback and perceived muscular effort. For amputees operating myo-controlled devices, this translates in a consistent delay between the grip of the object and the increase of the vertical lifting force. The ultimate aim of this study was to determine if the more reliable myoelectric interface from implanted electrodes, when combined with the incidental sensory feedback available to the subjects, could have any effect on the coupling of GF and LF. Surprisingly, eEMG prosthetic control worsened the grip and load force coordination, increasing the delay between them by 39% with respect to sEMG configuration (Fig. 6). This suggests that the incidental sensory feedback available was not rich enough for the subjects to rely on it and that, as predicted by Ernst et al. [34], they relied heavily on visual feedback in order to reduce the number of broken objects. We argue that, in the sEMG configuration, as the subjects were not able to modulate the GF accurately, they partially ignored the constraint of the fragile objects, successfully exploiting a feedforward grasping strategy. Even though one must not ignore that the subjects’ performance could potentially improve after intensive training, promoting the development of an internal model of the test object and task, these considerations are of relevant interest to confirm, in concert with previous studies [16, 17, 30, 35, 36], that a more reliable control interface (implanted myoelectric electrodes) and incidental feedback cannot compensate for the lack of tactile sensory feedback for the development and maintenance of the motor tasks internal model, and for the restoration of natural grasp behavior. Therefore, it is crucial to investigate how and if the situation would change when tactile feedback is reintroduced to close the usually open loop of prosthetic control. This result opens the path to a complementary study where the benefits of tactile sensory feedback via direct neural stimulation will be investigated.

## Conclusions

This study assessed the control of an osseointegrated prosthesis via implanted epimysial electrodes and compared this configuration to the conventional surface electrodes approach. The assessment was performed on a group of three transhumeral amputee subjects implanted with an osseointegrated e-OPRA Implant System that includes neuromuscular interfaces in addition to direct skeletal attachment. Grip force control and motor coordination were assessed in the context of delicate and routine grasp via the Virtual Eggs and the Pick and Lift tests. Results showed that implanted electrodes can provide a superior controllability over the prosthetic terminal device compared to conventional surface electrodes. Significant improvements were found in the control of the grip force and its reliability. However, these improvements failed to increase motor coordination, surprisingly worsening the relationship between grip and load forces observed in surface electrodes configuration. Even if proven functional and reliable, prosthetic control via implanted electrodes still depended highly on visual feedback. Therefore, even when a reliable human-machine interface is available, visual-auditory-osseoperceptive sensory feedback appeared insufficient for restoring natural grasp behavior in amputees. This result suggests the need for tactile sensory feedback to learn and maintain the motor tasks internal model. This study is relevant to the ongoing technological endeavor to develop neuromuscular interfaces aiming to direct neural stimulation for closed-loop prosthetic control, and towards the long-awaited evolution of neuroprosthetics for upper limbs.
